# Nutritional Composition and Biological Activities of Donkey Milk: A Narrative Review

**DOI:** 10.3390/foods14132337

**Published:** 2025-07-01

**Authors:** Qingyu Xu, Lin Wei, Xiuwen Chen, Hongzhen Zhu, Jinjin Wei, Mingxia Zhu, Muhammad Zahoor Khan, Changfa Wang, Zhenwei Zhang

**Affiliations:** College of Agriculture and Biology, Liaocheng University, Liaocheng 252059, China

**Keywords:** nutritional components, donkey–bovine milk comparison, biological activities, donkey milk applications, health promotion

## Abstract

Donkey milk has received increasing attention in recent years due to its unique nutritional composition and potential biological activities. This comprehensive review analyzed the main nutritional components of donkey milk, including proteins, lipids, carbohydrates, vitamins, and minerals, while also examining its significant biological activities such as antioxidant, antimicrobial, immunomodulatory, and anti-inflammatory properties. The protein profile of donkey milk is notable for its high proportion of whey proteins (55–65%), resembling human milk more closely than cow milk. Its relatively low-fat content (approximately 1.29%) with higher proportions of unsaturated fatty acids provides nutritional advantages for specific dietary needs. The carbohydrate content, primarily lactose, contributes to energy provision and calcium absorption. Donkey milk is also distinguished by its rich vitamin profile, particularly vitamin C (about 4.75 times higher than cow milk), and essential minerals including calcium, phosphorus, and zinc. The biological activities of donkey milk extend to various applications in infant nutrition, particularly for children with cow milk protein allergies, potential medical treatments for infections and inflammatory conditions, and cosmetic formulations. Despite these promising attributes, the donkey milk industry faces challenges including low milk yield, lack of standardized production methods, and quality control measures. The sustainable development of the donkey milk industry requires comprehensive approaches to resource protection, technological innovation, brand building, and supportive policies to realize its full potential in contributing to human health and economic development.

## 1. Introduction

The global donkey population stands at approximately 44 million, with an annual growth rate of about 1% [1,2]. The most significant increase has been observed in Sub-Saharan Africa, while the number of donkeys in Eastern Europe has decreased. China is one of the countries with the largest donkey populations, boasting a rich variety of breeds and approximately 30 different donkey species [2,3]. However, since 1991, the number of donkeys in China has been declining, from 9.444 million in 1996 to 2.593 million in 2016 [4]. By 2020, the majority of China’s donkeys were found in four provinces: Inner Mongolia (693,000 heads, accounting for 26.65% of the national total), Liaoning (401,000 heads, accounting for 15.43%), Xinjiang (361,000 heads, accounting for 13.90%), and Gansu [4,5]. The geographical distribution of donkey milk production closely correlates with these donkey population centers.

Regarding donkey milk production, due to the low milk yield of donkeys, which can produce only 1–2 L of milk per day and have a lactation period of about 6 months, the global donkey milk production is relatively limited [4,5]. However, with the growing popularity of health and wellness concepts worldwide, donkey milk, due to its unique nutritional value, such as a high proportion of whey protein (64.3% of total protein, significantly higher than traditional goat and cow milk) and its rich content of immunoglobulins, various vitamins, and minerals, aligns well with the trend of healthy consumption, and market demand is gradually expanding [6]. The global donkey milk market is experiencing significant growth, with a compound annual growth rate of about 9%, according to data from authoritative market research institutions. Behind this growth trend is the increasing demand for healthy dairy products among consumers. Donkey milk, with its unique nutritional components and potential health benefits, is gradually becoming a new hot spot in the dairy market [6]. As of 2019, Europe dominated production, accounting for nearly half of the market share. However, markets in the America and Asia Pacific regions are demonstrating rapid expansion [6]. While the cosmetics and personal care industries currently claim the largest share of the donkey milk market, analysts project that the food specialty industry will experience the most substantial increase in donkey milk consumption over the next five years [6,7,8]. China’s donkey milk industry remains in the early stages of industrialization, with a relatively underdeveloped industrial foundation. Regional market factors have created significant price disparities between eastern and western regions [7,8,9,10]. In regions such as Xinjiang, where animal husbandry is a primary economic activity, local herdsmen have realized substantial economic benefits from participation in the donkey dairy industry [11].

Currently, thousands of donkey milk farming bases are being established throughout China [7]. Despite the relatively low milk yield of donkeys compared with other dairy animals, the breeding return benefit is exceptionally high. The development of fresh donkey milk represents a mutually beneficial opportunity for all stakeholders in the donkey milk industry [7,8,9]. As a natural and organic food product, donkey milk not only offers rich nutritional content, but also contains various bioactive compounds with unique health benefits. Although donkey milk has been consumed for centuries in certain regions, it has recently gained significant attention due to its potential health applications [12,13]. Donkey milk represents both a valuable food resource and a product with broad application prospects, emerging as a topic of interest across multiple sectors [14,15].

**Figure 1:**
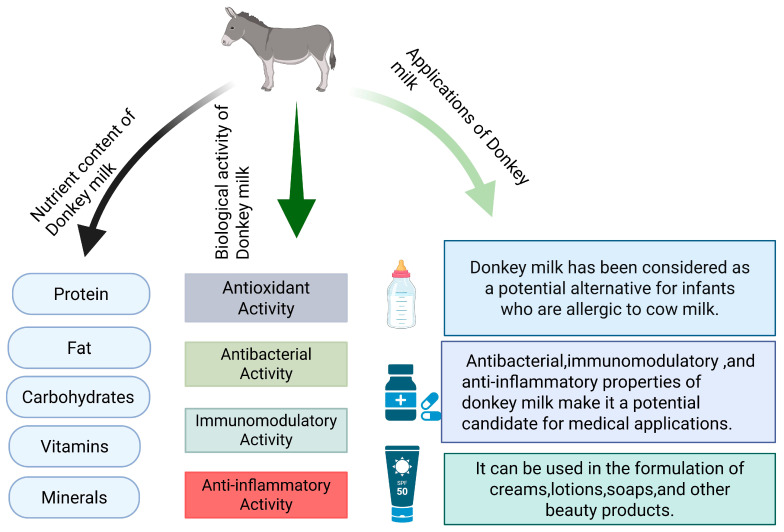
Bioactive components and health effects of donkey milk.

In recent years, donkey milk has attracted widespread scientific and commercial interest as a specialized animal dairy product. This paper is a narrative study, aiming to comprehensively describe the nutritional components and biological activity of donkey milk. Instead of using the strict evidence-based method of systematic review, this paper presents the current research status of the nutritional components and biological activity of donkey milk through sorting and summarizing the relevant literature. This review aimed to systematically review the research progress of the nutritional composition and bioactive components of donkey milk and deeply explore its health benefits. Research indicates that donkey milk contains abundant nutrients, including proteins, fats, carbohydrates, vitamins, and minerals, all of which contribute significantly to human health [16,17]. Additionally, donkey milk contains several specialized bioactive components, including growth factors, immunoglobulins, and antimicrobial peptides, which demonstrate anti-inflammatory, antioxidant, antibacterial, and anti-allergenic properties [18]. Consequently, donkey milk offers not only substantial nutritional value, but also important bioactive compounds that provide multiple health benefits (Figure 1) [19,20].

## 2. Literature Search Methodology

This review aimed to comprehensively outline the nutritional components and biological activities of donkey milk. During the literature screening process, we focused on studies directly related to the analysis of the nutritional components and biological activities of donkey milk. We prioritized research papers published in mainstream academic journals within the last five years to ensure timeliness and scientific accuracy. However, to provide comprehensive context, we also included selected articles published up to 2006.

The keywords used for the literature search were: donkey milk, nutritional composition, biological activities, and health benefits. Public databases, including PubMed, Web of Science, Google Scholar, and X-MOL, were utilized for the literature search. Only articles published in English and in internationally recognized SCI-indexed journals were considered for discussion. Theses, book chapters, conference papers, and unpublished data were excluded from this review.

## 3. Nutrient Content of Donkey Milk

### 3.1. Proteins

Donkey milk contains a relatively high protein content, ranging from 1.4 to 1.8 g/100 mL, primarily consisting of whey proteins and caseins [21]. A distinctive characteristic of donkey milk protein is the high proportion of whey protein, which accounts for approximately 55–65% of the total protein content. This proportion more closely resembles human breast milk than cow milk. Whey protein, rich in essential amino acids, is often referred to as the “king of proteins” [22] and represents a fundamental component for human growth, development, and anti-aging processes. The principal whey proteins in donkey milk include α-lactalbumin, β-lactoglobulin, and lactoferrin [23]. α-Lactalbumin serves as an essential component that not only provides a rich source of essential amino acids such as leucine and isoleucine, but also plays a crucial role in lactose synthesis. It demonstrates high biological value and digestibility [24,25,26]. Although present in lower concentrations compared with other milk sources, β-lactoglobulin still contributes significantly to the overall protein profile [23].

**Figure 2:**
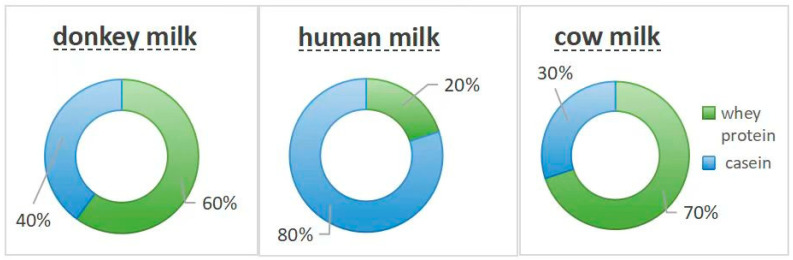
The proportion of whey protein and casein content in donkey, human, and cow milk.

Lactoferrin represents a multifunctional protein with antimicrobial, antiviral, and immunomodulatory activities [27]. Its iron-binding capacity inhibits the growth of iron-dependent bacteria and fungi. Traditional classification categorizes milk as either casein milk or whey protein milk based on the protein composition [27,28]. Casein constitutes approximately 80% of cow milk protein, 50% of donkey milk protein, and less than 50% of human breast milk protein. Whey proteins demonstrate both antibacterial and anti-inflammatory properties [28]. Donkey milk contains a diverse array of essential amino acids similar to human breast milk, such as lysine, methionine, threonine, etc., facilitating efficient protein absorption and utilization [25,26]. These characteristics establish donkey milk as an ideal protein source, particularly appropriate for populations with increased protein requirements including children, pregnant women, and lactating mothers (Figure 2) [28,29].

### 3.2. Lipids

Donkey milk is characterized by its relatively low-fat content, averaging about 1.29%, which is only about 40% of the fat content in regular cow’s milk [30]. The primary component of its lipid profile is triglycerides (TAGs), which make up over 95% of the total fat content. This high proportion of triglycerides allows for the rapid release of fatty acids during digestion, making it easier for the human body to absorb [30]. Additionally, donkey milk contains a small amount of diacylglycerol (DAGs), which has health benefits, such as reducing fat accumulation and regulating blood lipids, thereby enhancing the nutritional value of donkey milk [30].

Although donkey milk contains less phospholipids than cow’s milk, it still plays a significant role in its lipid system [31]. Phospholipids are essential components of cell membranes and are involved in various physiological activities in the human body, promoting fat metabolism and supporting the normal functioning of the nervous system [31]. Additionally, donkey milk has a lower cholesterol content, which aligns with its overall low-fat profile, further reducing the impact on blood lipids after consumption [31]. Furthermore, donkey milk contains comparatively lower levels of saturated fatty acids. Since the excessive consumption of saturated fatty acids has been associated with increased cardiovascular disease risk, the reduced saturated fatty acid content in donkey milk represents a significant nutritional advantage [32].

In contrast, donkey milk features a higher proportion of unsaturated fatty acids, particularly polyunsaturated fatty acids, which are essential for human health [32]. These include important compounds such as linoleic acid and linolenic acid. Linoleic acid, which cannot be synthesized by the human body and must be obtained through dietary sources, is relatively abundant in donkey milk and constitutes a higher percentage of total fat compared with other animal milks [32]. Research has demonstrated that linoleic acid can effectively regulate blood lipids, reduce cholesterol levels, and help prevent cardiovascular diseases [31,32]. Additionally, donkey milk contains measurable amounts of conjugated linoleic acid, which performs various physiological functions including lipid metabolism regulation, serum cholesterol reduction, blood lipid lowering, blood vessel elasticity improvement, blood pressure reduction, microcirculation enhancement, and atherosclerosis inhibition [33]. Consequently, conjugated linoleic acid is often referred to as a “vascular scavenger” and has demonstrated beneficial effects in the prevention and auxiliary treatment of cardiovascular diseases and obesity [33]. The triglyceride structure and composition in donkey milk also exhibit distinctive characteristics. The fatty acid distribution within its triglyceride molecules likely differs from that of other animal milks, potentially influencing its digestibility, absorption properties, and physiological functions [33]. For instance, donkey milk contains relatively high levels of medium-chain triglycerides, which are characterized by their small molecular size, enhanced digestibility and absorption, and rapid energy provision [34].

**Figure 3:**
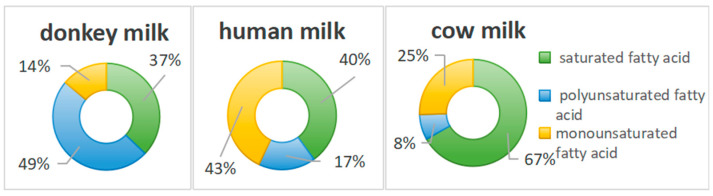
The proportion of fatty acid content in donkey, human, and cow milk.

Due to its unique lipid composition, donkey milk offers numerous health benefits [34]. Although donkey milk contains fewer fatty acids than human breast milk, indicating a lower energy content, this reduced fat profile makes it an appropriate choice for individuals needing to monitor their fat intake [35]. The combination of low-fat content and high unsaturated fatty acid levels contributes to reduced cardiovascular disease risk by minimizing cholesterol deposition in the blood vessel walls, thereby supporting cardiovascular health [35]. For infants and young children, donkey milk lipids provide essential energy and nutrients required for growth and development while promoting brain and nervous system development. Moreover, the unsaturated fatty acids in donkey milk help maintain intestinal flora balance by stimulating beneficial bacterial growth and supporting normal intestinal function (Figure 3) [34,35].

### 3.3. Carbohydrates

Carbohydrates in donkey milk are predominantly present as lactose. The average lactose content per 100 mL of donkey milk is approximately 6.34 g [36]. Lactose, a disaccharide, requires enzymatic hydrolysis into glucose and galactose in the human gastrointestinal tract before absorption and utilization can occur [36]. Carbohydrates constitute an important energy source in donkey milk. According to the energy conversion coefficient of food nutrients, each gram of carbohydrate can provide 17 kJ of energy [36]. Following digestion and hydrolysis, lactose in donkey milk can provide substantial energy for the human body and maintain normal physiological functions. Notably, lactose can form complexes with minerals such as calcium, thereby enhancing calcium absorption [37]. For individuals requiring calcium supplementation, the lactose in donkey milk significantly improves the bioavailability of calcium. Concurrently, lactose serves as a substrate for probiotic microorganisms in the intestinal tract, promoting the proliferation and growth of beneficial bacteria, thus contributing to intestinal microbiota balance and gastrointestinal health [37].

**Figure 4:**
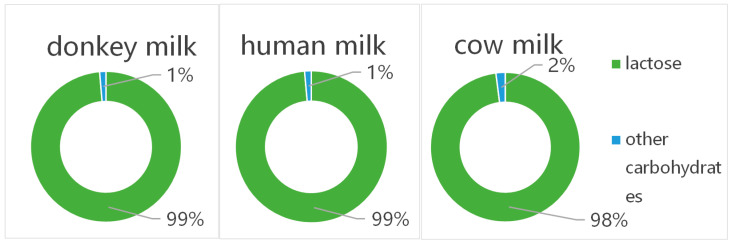
The ratio of donkey, cow, and human milk from lactose to total carbohydrates (per 100 g).

The relatively high lactose content in donkey milk may have implications for lactose intolerant individuals, although some studies have suggested better tolerability than bovine milk in certain cases due to its unique compositional profile [37]. However, individuals with lactase deficiency may be unable to fully digest and absorb the lactose in donkey milk, potentially experiencing symptoms of lactose intolerance such as abdominal distension, pain, and diarrhea after consumption [36,37]. For diabetic patients, despite the relatively high carbohydrate content in donkey milk, its impact on blood glucose levels may be attenuated due to its low fat and protein content [36,37]. Nevertheless, diabetic patients should exercise caution when consuming donkey milk, with particular attention to portion control and the close monitoring of glycemic responses (Figure 4) [36,37]. Table 1 presents a comparative analysis of the compositions of donkey, cow, and human milk.

**Table 1:** Summary table of the protein, fat, and carbohydrate content of donkey, cow, and human milk.

Milk Compositions	Donkey Milk	Cow Milk	Human Milk
Protein (g/kg)	12.5–21.8	31.0–38.0	9.0–17.0
Fat (g/kg)	1.0–14.0	35.0–39.0	35.0–40.0
Lactose (g/kg)	60.3–72.8	44.0–49.0	63.0–70.0
Casein (g/kg)	6.4–10.3	24.6–28.0	3.2–4.2
Lactoalbumin (g/kg)	4.9–9.3	5.5–7.0	6.8–8.3

The data presented in this table were derived from the following publications [15,17,24,32].

### 3.4. Vitamins

Donkey milk represents an excellent source of various vitamins, with a notably high vitamin C content, approximately 4.75 times that of bovine milk [37,38]. Vitamin C functions as a potent antioxidant, capable of scavenging free radicals in the body, thereby reducing oxidative stress-induced cellular damage and exhibiting antioxidant and anti-aging effects. Additionally, it promotes collagen synthesis, contributing to the maintenance of normal structure and function of the skin, blood vessels, bones, and other tissues, and plays a significant role in wound healing processes [38].

Donkey milk also contains appreciable amounts of vitamin A, which is crucial for maintaining normal epithelial cell function [38]. Vitamin A helps preserve the health and integrity of epithelial tissues in the skin, respiratory tract, and digestive system, preventing conditions such as skin dryness and keratinization. Moreover, it plays a key role in protecting ocular health and maintaining optimal vision, preventing conditions such as night blindness and dry eye disease [38]. Furthermore, donkey milk contains a spectrum of B vitamins including B1, B2, B6, and B12. These vitamins serve as essential coenzymes in energy metabolism, facilitating the conversion of dietary carbohydrates, lipids, and proteins into bioavailable energy, thereby maintaining normal physiological functions [38,39]. For instance, vitamin B1 alleviates fatigue by inhibiting cholinesterase activity, while vitamin B12 is indispensable for normal neurological development and functional maintenance, with deficiency potentially leading to neurological disorders [39].

**Figure 5:**
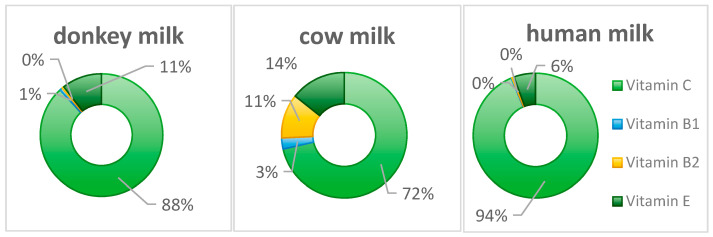
The proportion of vitamin content in donkey, cow, and human milk.

Although the vitamin D content is not exceptionally high, it is nevertheless significant for calcium absorption and utilization [40]. Vitamin D promotes intestinal calcium and phosphorus absorption, maintains physiological blood calcium and phosphorus levels, contributes to healthy bone and dental development, and plays a beneficial role in preventing skeletal disorders such as rickets and osteoporosis [40]. Vitamin E in donkey milk also functions as an antioxidant, protecting cells from free radical damage and working synergistically with vitamin C to enhance the body’s antioxidant capacity. Vitamin E also contributes to maintaining normal reproductive system function (Figure 5) [39,40].

### 3.5. Minerals

Donkey milk contains essential minerals including calcium, phosphorus, potassium, sodium, iron, and zinc. The calcium content of donkey milk (80–120 mg/100 g) is significantly lower than that of bovine milk (120–130 mg/100 g), but substantially higher than human milk (32–35 mg/100 g) [41]. Calcium, a major constituent of human bone and dental tissue, is essential for maintaining skeletal strength and density. Additionally, calcium plays a pivotal role in various physiological processes including muscle contraction, neuronal signal transduction, and blood coagulation [41]. The calcium in donkey milk exhibits high bioavailability and contributes to the prevention of skeletal disorders such as osteoporosis, particularly benefiting skeletal development in children and bone health maintenance in the elderly [41]. Moreover, phosphorus participates in intracellular energy metabolism, including adenosine triphosphate (ATP) synthesis, and is critical for cellular membrane composition and stability, ensuring normal cellular physiological function [41].

**Figure 6:**
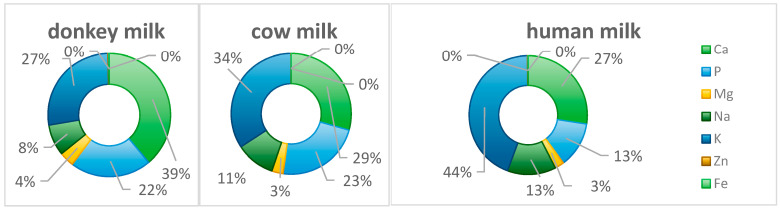
The proportion of mineral content in donkey, cow, and human milk.

Potassium in donkey milk is essential for maintaining electrolyte homeostasis. Potassium ions participate in neuronal impulse conduction and regulate muscular contraction, contributing to normal cardiac rhythm [41]. Intracellularly, potassium functions as the primary cation and plays a crucial role in cellular osmotic balance, ensuring normal cellular morphology and function. In conjunction with sodium, potassium regulates osmolality and acid–base balance while modulating neuromuscular excitability [41]. A moderate sodium intake is necessary for normal physiological function, although excessive consumption may lead to hypertension and related disorders; the sodium content in donkey milk falls within physiologically appropriate parameters [41]. Donkey milk contains appreciable iron, with concentrations 2–3 times higher (0.3–0.5 mg/100 g) than bovine milk. Iron is a critical component for hemoglobin synthesis and is essential for oxygen transport and storage [42]. Iron deficiency can result in anemia, adversely affecting energy metabolism and immune function. Although the iron content in donkey milk is moderate, it provides a complementary contribution to dietary iron intake [43]. Additionally, donkey milk contains zinc, with significantly higher concentrations (0.5–0.7 mg/100 g) than human milk. Zinc participates in the synthesis and activation of numerous enzymes and significantly influences growth and development, immune regulation, and gustatory and olfactory function [43]. During childhood and adolescence, zinc exerts substantial effects on physical and cognitive development and is crucial for normal reproductive function and dermatological health. Donkey milk contains appropriate concentrations of these minerals, enhancing its overall nutritional profile (Figure 6) [43,44]. The Table 2 presents the nutritional compositions including vitamins, amino acids, minerals and proteins of donkey milk.

**Table 2:** Nutritional composition of donkeymilk (vitamins, minerals, amino acids, and proteins).

Nutritional Classification	Specific Ingredients	Content
Vitamin	Ve	4.49 mg/kg
	Vb1	4.44 μg/100 g
	Vb2	6.07 μg/100 g
	Vc	5.16 mg/kg
Minerals	Ca	590 mg/kg
	P	513 mg/kg
	K	438 mg/kg
	Na	194 mg/kg
	Mg	47.6 mg/kg
	Zn	1.4 mg/kg
	Fe	2.1 mg/kg
Amino acids	Asp	0.12%
	Thr	0.06%
	Ser	0.07%
	Glu	0.24%
	Gly	0.02%
	Ala	0.04%
	Cys	0.04%
	Val	0.07%
	Pro	0.11%
	Mct	0.05%
	Ilc	0.06%
	Leu	0.12%
	Tyr	0.05%
	Phe	0.10%
	Lys	0.11%
	His	0.04%
	Arg	0.08%
Proteins	CN	6.11 g/kg
	WP	8.63 g/kg
	α-LA	2.00 g/kg
	β-LG	3.46 g/kg
	SA	0.43 g/kg
	Ig	0.16 g/kg
	LF	0.33 g/kg
	LYS	2.25 g/kg

This table presents data extracted from the following referenced publications [15,17,24,32,33,40].

## 4. Biological Activity of Donkey Milk

### 4.1. Antioxidant Activity

Donkey milk contains several antioxidant compounds including vitamin C, vitamin E, and various enzymatic antioxidants [45]. Vitamin C functions as a potent antioxidant through the direct scavenging of reactive oxygen species (ROS), particularly superoxide anion radicals and hydroxyl radicals. Additionally, it participates in the regeneration of oxidized vitamin E, thereby enhancing the overall antioxidant defense system [45]. Vitamin E, primarily localized in cell membranes, prevents the free radical-mediated oxidation of unsaturated fatty acids, thus maintaining membrane integrity and stability [46]. Superoxide dismutase (SOD), a significant enzymatic antioxidant in donkey milk, catalyzes the dismutation of superoxide radicals into hydrogen peroxide and molecular oxygen, constituting the initial defensive mechanism against oxidative damage [46]. This enzyme represents the first line of cellular protection against oxidative stress.

The antioxidant properties of donkey milk extend to its bioactive peptides and proteins. Certain peptides exhibit antioxidant activity through metal ion chelation, reducing the potential for metal-catalyzed oxidation reactions [47]. Lactoferrin, beyond its established antimicrobial properties, demonstrates considerable antioxidant capacity through various mechanisms, protecting cells from oxidative stress-induced damage. Furthermore, conjugated linoleic acid and related compounds in donkey milk exhibit antioxidant characteristics, effectively reducing lipid peroxidation [47].

The antioxidant components in donkey milk interact directly with free radicals, converting them to more stable compounds [48]. For instance, vitamins C and E donate hydrogen atoms to reduce free radicals while becoming oxidized themselves. Antioxidant peptides neutralize free radicals by electron donation, inhibiting the propagation of radical chain reactions and mitigating free radical damage to biological macromolecules including DNA, proteins, and lipids [49]. Some components chelate metal ions such as iron and copper, which typically catalyze free radical production through reactions like the Fenton reaction, where iron ions react with hydrogen peroxide to generate highly reactive hydroxyl radicals. By chelating these metal ions, the antioxidant peptides in donkey milk indirectly reduce free radical generation through decreased catalytic activity [48,49].

**Figure 7:**
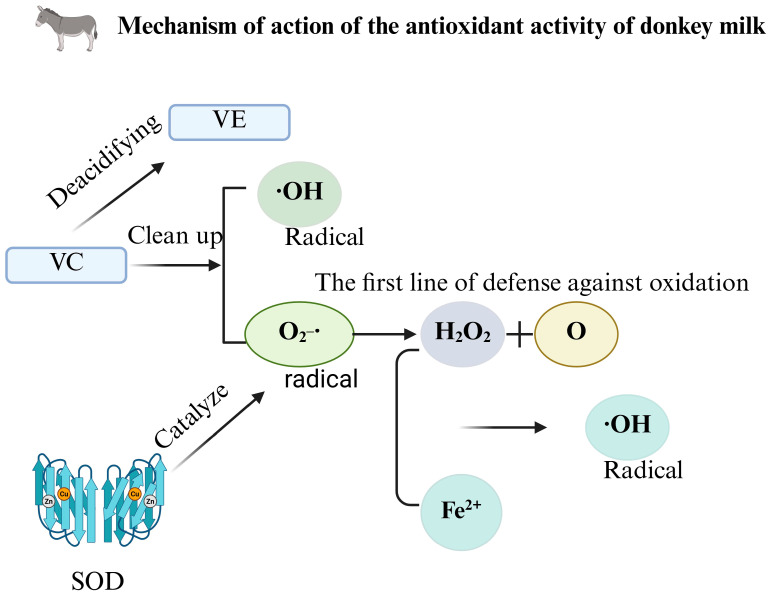
Mechanism of the antioxidant activity of donkey milk.

Certain components of donkey milk may enhance the antioxidant capacity via modulation of intracellular antioxidant enzyme expression and activity [49]. These bioactive compounds potentially activate the transcription of antioxidant enzyme genes, increasing the synthesis of SOD, catalase, and glutathione peroxidase, thus improving the organism’s capacity to eliminate free radicals [49]. The aging process is intimately associated with oxidative stress. The antioxidant constituents of donkey milk may delay cellular and tissue aging by scavenging free radicals and reducing oxidative damage [49]. For example, by decreasing oxidative damage to collagen and elastin fibers in skin cells, these compounds help maintain skin elasticity and appearance, reducing wrinkle formation and potentially mitigating skin aging [49]. Chronic oxidative stress contributes to the pathogenesis of numerous chronic diseases including cardiovascular disease, cancer, and diabetes. The antioxidant activity of donkey milk may reduce systemic oxidative burden, decrease free radical-mediated damage to vascular endothelial cells, and reduce atherosclerosis risk [50]. The protective effects on cellular DNA may partially reduce the cancer incidence, while the maintenance of normal pancreatic β-cell function has potential significance for diabetes prevention and management (Figure 7) [50,51].

### 4.2. Antibacterial Activity

Lactoferrin represents a significant antimicrobial component in donkey milk, primarily functioning through its high iron-binding capacity [52]. By chelating iron ions, lactoferrin restricts an essential nutrient required for bacterial metabolism, thereby inhibiting microbial growth and proliferation. This protein demonstrates antimicrobial efficacy against diverse Gram-positive and Gram-negative bacteria including *Escherichia coli* and *Staphylococcus aureus* [53]. The iron-sequestration mechanism specifically targets bacterial iron-dependent metabolic pathways; for instance, iron-containing enzymes in bacterial respiratory chains exhibit reduced activity under iron-limited conditions, resulting in growth inhibition.

Immunoglobulins present in donkey milk contribute to its antimicrobial properties through multiple mechanisms [53,54]. These proteins specifically recognize and bind to bacterial surface antigens, neutralize bacterial toxins, and prevent bacterial adhesion to host cells. Additionally, immunoglobulins activate the complement system, enhancing immune defense and promoting phagocyte-mediated bacterial clearance [54,55]. The immunomodulatory components in donkey milk stimulate macrophage phagocytic activity and lymphocyte-mediated immune responses, facilitating more efficient bacterial elimination. Furthermore, these components promote moderate inflammatory responses, attracting immune cells to infection sites and augmenting antibacterial capacity [54,55].

Lysozyme constitutes another critical antimicrobial factor in donkey milk. This enzyme hydrolyzes peptidoglycan in the bacterial cell walls, compromising structural integrity and inducing bacterial lysis due to osmotic pressure imbalance [56,57]. Lysozyme demonstrates a particular efficacy against pathogenic bacteria with peptidoglycan-rich cell walls. The bactericidal mechanism involves the direct enzymatic degradation of structural components, representing a classical direct antimicrobial action [57,58].

Various antimicrobial peptides in donkey milk exert their effects by disrupting bacterial membrane structures and interfering with metabolic processes [59]. These small molecular peptides, along with lysozyme, directly target bacterial cellular components, causing structural disruption, cytoplasmic leakage, and the consequent loss of cellular function [59]. The oligosaccharides in donkey milk function as prebiotics, selectively promoting the growth of beneficial intestinal bacteria such as *Bifidobacterium* and *Lactobacillus* species. These beneficial microorganisms competitively inhibit pathogenic bacteria through nutrient competition and the production of antimicrobial substances, maintaining intestinal microecological balance and reducing infection risk [59].

**Figure 8:**
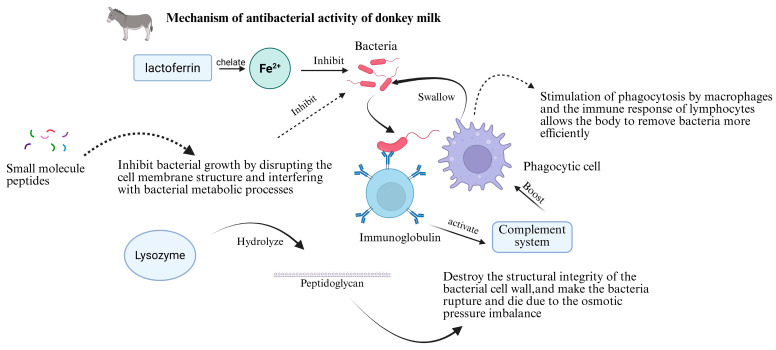
Mechanism of the antibacterial activity of donkey milk.

In the gastrointestinal tract, donkey milk’s antimicrobial activity inhibits pathogenic bacterial proliferation, potentially preventing and treating gastrointestinal infections [59]. For instance, in cases of diarrhea caused by *E. coli* or *Salmonella*, the antimicrobial components may alleviate symptoms and shorten disease duration while promoting intestinal microbiota stability and overall intestinal health [60]. Donkey milk also demonstrates potential topical antimicrobial applications. Skincare products containing donkey milk components may inhibit surface bacterial growth, potentially providing relief for bacterial skin infections such as acne and furuncles while maintaining skin health and cleanliness [60]. Regarding respiratory tract infections, although donkey milk cannot directly act upon the respiratory tract, its consumption may modulate systemic immunity and enhance antimicrobial capacity, potentially reducing respiratory infection symptoms and preventing infection onset. This may occur through enhanced immune defense functions of the respiratory mucosa and reduced bacterial colonization in the respiratory tract (Figure 8) [60].

### 4.3. Immunomodulatory Activity

Donkey milk contains a diverse array of immunomodulatory components, with immunoglobulins, particularly IgG, representing significant bioactive constituents [61]. These immunoglobulins recognize and bind to pathogenic organisms, including bacteria and viruses, thereby initiating immune defense mechanisms and neutralizing pathogen virulence factors, consequently enhancing host resistance to infection. Additionally, donkey milk contains various bioactive peptides with immunomodulatory properties that influence immune cell function through multiple signaling pathways [61].

Lactoferrin, abundant in donkey milk, exhibits multifaceted immunomodulatory functions. Beyond its iron-sequestration capability, lactoferrin regulates immune cell activity by promoting macrophage phagocytic function and enhancing lymphocyte proliferation and differentiation, thereby modulating overall immune status [62]. This protein interacts with specific receptors on macrophage surfaces, triggering intracellular signal transduction cascades that stimulate the secretion of immunoregulatory factors, including cytokines and chemokines, which subsequently initiate immune defense responses [62].

Lysozyme represents another critical immunomodulatory component in donkey milk. While its bactericidal activity through peptidoglycan hydrolysis is well-established, lysozyme also stimulates immune cell activity and participates in immune response regulation [62]. Furthermore, oligosaccharides present in donkey milk function as prebiotics, promoting beneficial intestinal microbiota growth and indirectly influencing gut-associated immune function [62]. The immunomodulatory properties of donkey milk hold particular significance for specific population groups. In infants and young children with immature immune systems, the immunoactive constituents in donkey milk may enhance resistance to pathogens and reduce infectious disease incidence. For example, immunoglobulins provide passive immune protection against enteric pathogen invasion, potentially supporting healthy development in pediatric populations [63]. In elderly individuals experiencing age-associated immune decline, donkey milk may serve as a nutritional supplement for immune function modulation [62,63]. Its immunomodulatory components potentially enhance immune defense capabilities in the elderly, reducing infection risk, improving quality of life, and possibly preventing or mitigating chronic diseases associated with immunosuppression [63].

**Figure 9:**
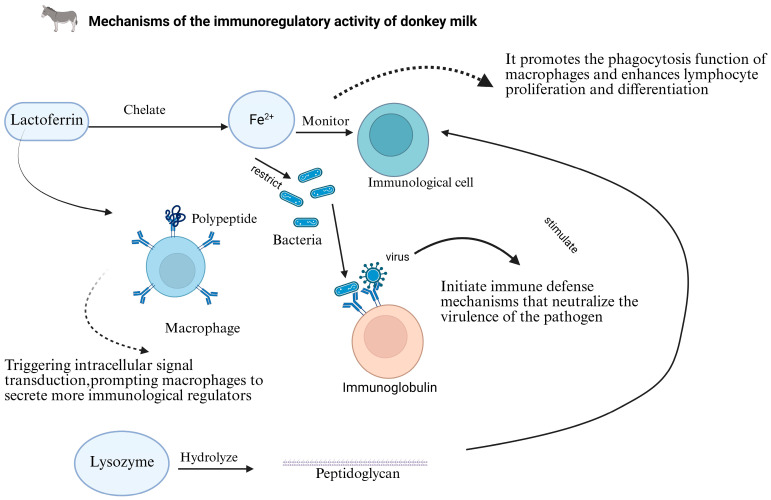
Mechanisms of the immunoregulatory activity of donkey milk.

For immunocompromised populations, such as cancer patients undergoing chemotherapy or individuals with primary immunodeficiency disorders, the immunoregulatory activity of donkey milk may contribute to improved immune status [63]. While not a replacement for conventional therapeutic interventions, donkey milk might function as an adjunctive approach to reduce complication incidence, particularly infections associated with compromised immunity (Figure 9) [61,62,63].

### 4.4. Anti-Inflammatory Activity

Proteins and peptides in donkey milk exhibit significant anti-inflammatory activity [64]. Lactoferrin plays a crucial role in this process by chelating iron ions, thereby limiting the iron required for bacterial growth and inhibiting inflammatory responses related to bacterial infection. Additionally, various small molecule peptides may participate in regulating immune cell activity and reducing inflammatory mediator release [64]. The unsaturated fatty acids present in donkey milk, particularly conjugated linoleic acids, demonstrate anti-inflammatory properties through modulation of inflammatory signaling pathways, inhibition of proinflammatory factor production (including tumor necrosis factor-α and interleukin-6), and reduction of inflammatory responses [65].

Lysozyme and other components in donkey milk contribute significantly to the anti-inflammatory process [56,57]. Lysozyme effectively destroys bacterial cell walls, thereby reducing inflammation caused by bacterial infection and potentially exerting regulatory effects on inflammatory cell activity [66]. Donkey milk regulates immune system balance by promoting the differentiation and function of anti-inflammatory immune cells, such as increasing regulatory T cell numbers and activity, while simultaneously inhibiting pro-inflammatory immune cell overactivation (e.g., Th17 cells), thus attenuating inflammatory response intensity. The bioactive components in donkey milk interfere with inflammation-related signaling pathways, notably the NF-κB pathway [67]. Through inhibition of NF-κB activation, donkey milk reduces the expression of numerous downstream proinflammatory genes, including inflammatory factors and adhesion molecules, thereby mitigating inflammatory response-induced tissue damage [67].

**Figure 10:**
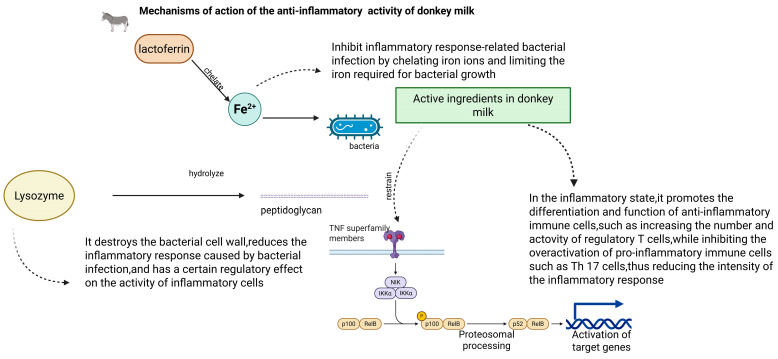
Mechanism of the anti-inflammatory activity of donkey milk.

In gastrointestinal inflammation, donkey milk’s anti-inflammatory activity reduces inflammatory damage to intestinal mucosa [68]. Its constituents facilitate repair of damaged intestinal mucosa, inhibit harmful gut bacteria proliferation, decrease inflammatory factor stimulation in the gut, and alleviate inflammatory symptoms such as abdominal pain and diarrhea [69]. Regarding respiratory tract inflammation, donkey milk may inhibit respiratory tract pathogen growth and regulate immune responses, thereby reducing inflammation-induced symptoms such as cough and asthma. Specifically, its anti-inflammatory components can decrease respiratory mucosal congestion and edema, thereby improving respiratory function. Furthermore, donkey milk demonstrates potential applications in treating skin inflammation [69,70]. Either topical application or consumption of donkey milk may ameliorate skin inflammation, reduce skin redness and itching, promote skin repair and regeneration, and potentially provide adjunctive therapeutic effects for various skin inflammatory conditions, including allergic dermatitis and eczema (Figure 10) [71].

## 5. Applications of Donkey Milk

Donkey milk has demonstrated unique value in various fields. In infant nutrition, its composition closely mirrors that of breast milk, particularly in terms of protein and nutritional content [72,73,74]. It can serve as a viable alternative for infants with allergies to cow’s milk, providing essential nutrients necessary for infant growth and development [75,76,77,78]. Moreover, it is more easily digested by sensitive infants, though it may need to be fortified to meet the full nutritional needs of infants [79,80,81]. In medical applications, donkey milk, due to its antibacterial, immune-regulating, and anti-inflammatory properties, holds significant potential in treating infections, immune-related diseases, and inflammatory conditions. For instance, the topical application of donkey milk-based products may provide therapeutic benefits for skin infections and wounds due to its antibacterial and anti-inflammatory effects. Furthermore, donkey milk shows promise in managing gastrointestinal disorders by potentially improving the gut microbiota and modulating intestinal immune responses [82]. In cosmetics, donkey milk, rich in nutrients, possesses antioxidant and moisturizing properties, making it an ideal ingredient in cosmetic formulations such as creams, lotions, and soaps [83,84]. The proteins and lipids present in donkey milk contribute to skin nourishment, enhanced elasticity, and reduced wrinkle appearance [85,86]. Additionally, its antioxidant components provide protection against oxidative damage induced by environmental factors including UV radiation and pollution [87].

## 6. Challenges and Future Perspectives

Despite its numerous potential benefits, the donkey milk industry faces significant challenges in production, processing, and commercialization. A primary constraint is the relatively low milk production in donkeys, resulting in elevated production costs and limited supply. Furthermore, there exists a deficiency in standardized production methods and quality control measures, potentially compromising the product quality consistency [88,89,90,91]. To promote sustainable development within the donkey milk industry, multiple aspects require attention. Resource protection and rational utilization are paramount. As the industrial foundation, donkey health directly influences the milk quality and yield. Consequently, proper attention must be directed toward feeding management, disease prevention, and nutrition, while reasonably planning donkey populations to prevent overdevelopment [92,93].

Technological innovation and research development represent crucial factors. In-depth investigations into the nutritional composition and biological activity of donkey milk will facilitate targeted product development, while an exploration of the processing technologies and preservation methods can enhance the added value and competitive advantage [94,95]. Establishing local characteristic brands may improve consumer awareness and trust, with modern marketing strategies strengthening promotion, expanding distribution channels, and increasing the market share. Concurrently, governmental support through policy formulation could promote healthy industry development, while strengthening the cooperation between upstream and downstream enterprises within the industrial chain could foster an integrated model that reduces costs and improves efficiency [96].

Future research endeavors should further explore donkey milk’s potential health benefits through intensive studies of its biological activity, the optimization of processing methods that preserve nutrients and bioactive ingredients, and the development of reliable quality control standards [97,98]. Additionally, efforts to improve donkey milk production efficiency through enhanced breeding and management practices will be essential to increase accessibility and affordability for consumers [98,99].

## 7. Conclusions

Donkey milk represents a unique and valuable food resource with an exceptional nutritional composition and biological activities that more closely resemble human milk than cow milk. Its high proportion of whey proteins (55–65%), favorable fatty acid profile, abundant vitamins (particularly vitamin C at levels 4.75 times higher than in cow milk), and essential minerals contribute significantly to its potential applications in infant nutrition, medical treatments, and cosmetic formulations. The documented antioxidant, antimicrobial, immunomodulatory, and anti-inflammatory properties further enhance its value as a functional food, offering considerable benefits for various demographic groups including infants with cow milk allergies, elderly individuals experiencing immunosenescence, and people with specific health conditions.

Despite these promising attributes, the donkey milk industry faces significant challenges that require comprehensive solutions for sustainable development. To address issues such as low milk yield, lack of standardized production methods, and inadequate quality control measures, future efforts should focus on implementing scientific breeding programs and optimized feeding management, developing standardized production protocols, conducting more in-depth clinical research on health benefits, investing in preservation technologies for bioactive components, establishing reliable quality standards, increasing consumer education, encouraging supportive governmental policies, and implementing measures to protect donkey genetic resources. Through this balanced approach combining scientific research, technological advancement, and sustainable resource management, the donkey milk industry can overcome the current limitations and realize its full potential in contributing to human health and economic development, particularly in regions with significant donkey populations.

## Data Availability

No new data were created or analyzed in this study. Data sharing is not applicable to this article.
